# Genome-Wide Association Study on Lodging Resistance-Related Traits in Oats

**DOI:** 10.3390/plants15060861

**Published:** 2026-03-11

**Authors:** Lijun Zhao, Rui Yang, Yantian Deng, Xiaopeng Zhang, Lijun Shi, Bai Du, Mengya Liu, Junmei Kang, Xiao Li, Tiejun Zhang

**Affiliations:** 1 School of Grassland Science, Beijing Forestry University, Beijing 100083, China; zlj202309@bjfu.edu.cn (L.Z.); yangrui0206@bjfu.edu.cn (R.Y.); dengyantian2022@163.com (Y.D.); 15025837939@163.com (X.Z.); slj0925@bjfu.edu.cn (L.S.); du17325370629@bjfu.edu.cn (B.D.); lmy0214@bjfu.edu.cn (M.L.); 2 Institute of Animal Science, Chinese Academy of Agricultural Sciences, Beijing 100193, China; kangjunmei@caas.cn

**Keywords:** *Avena sativa* L., lodging resistance, genome-wide association study (GWAS), quantitative trait nucleotides (QTNs), candidate genes

## Abstract

Oat (*Avena sativa* L.), as an essential dual-purpose grain and forage crop, exhibits lodging resistance as a key factor directly impacting yield and quality. Therefore, breeding new oat varieties with lodging resistance is important to increase crop productivity and economic benefits. Using 130 oat germplasm as materials, 7 lodging resistance-related traits of oat, including plant height (PH), the fresh weight of single stem (FWSS), the length of basal second internode (LBSI), diameter of basal second internode (DBSI), wall thickness of basal second internode (WTBSI), stem breaking strength (SBS), and stalk puncture strength (SPS), were investigated in two experimental sites for one year. The results indicate that the seven lodging resistance-related traits exhibit a continuous distribution overall and generally follow a typical distribution pattern. A total of 36,928,068 high-quality Single-nucleotide polymorphisms (SNPs) generated from whole-genome resequencing were used for genome-wide association study (GWAS). Based on the BLINK (Bayesian-information and Linkage-disequilibrium Iteratively Nested Keyway) model threshold (−log_10_(P) ≥ 6), 379 quantitative trait nucleotides (QTNs) associated with lodging resistance-related traits were identified. Among them, 38, 34, 78, 66, 55, 18, and 94 QTNs were associated with PH, FWSS, SBS, SPS, LBSI, DBSI, and WTBSI, respectively. Notably, three QTNs associated with FWSS and one QTN associated with SBS were stably detected across both environments, representing valuable markers for molecular breeding. From these loci, 54 candidate genes were annotated. Ranked by the number of candidate genes per trait, LBSI contained the highest number (14), followed by WTBSI (12), SPS (11), SBS (7), PH (5), and FWSS (5). Our findings provide critical support for analyzing the genetic mechanism of oat lodging resistance. Moreover, this study also offers a material and theoretical basis for the subsequent development of molecular markers and the breeding of new lodging-resistant oat varieties.

## 1. Introduction

Oat (*Avena sativa* L.), a cereal species within the genus Avena, is an important crop cultivated for both food and feed [1]. It is highly valued as animal feed due to its excellent palatability, digestibility, and storage stability [2]. However, lodging is a key limiting factor affecting oat yield and quality [3]. In particular, frequent rainfall and strong winds after heading often induce severe lodging, which impairs mechanized harvesting and reduces both the yield and quality of forage. Consequently, lodging has become a major constraint limiting the achievement of high yield and quality in oats [4]. Therefore, improving lodging resistance is a key breeding objective in oat improvement programs.

Previous studies have demonstrated that lodging is influenced by the genetic basis governing plant stem mechanical properties and related traits, such as plant height, stem weight, internode length, and stem wall thickness [5]. For instance, analysis of two doubled-haploid wheat populations revealed that natural lodging was most strongly correlated with plant height, followed by stem diameter and stem wall thickness [6]. Other research has indicated that a greater stem diameter provides structural support and strength to the plant, and a thicker stem can better withstand external pressures [7]. Stem puncture strength (SPS) and stem breaking strength (SBS) are key indicators for directly measuring stem mechanical properties [8]. SPS reflects the resistance of the stem wall to penetration, while an increased SBS indicates a stronger and more rigid stem at the lower part of the plant [9].

Genome-wide association study (GWAS) serves as an effective tool for deciphering the genetic mechanisms underlying complex traits [10]. It has been used in the identification of genes controlling key breeding traits, including yield [11], grain quality [12], and stress tolerance [13] in various crops, such as maize [14] and wheat [15]. With the rapid advancement of bioinformatics, statistics, and genomics, GWAS has been widely applied in early-stage molecular breeding to identify trait-associated markers. For example, He et al. [16] performed a GWAS on 895 barley accessions and identified 113 genes related to flowering time. Similarly, Zhang et al. [17] conducted a GWAS using 251 spring wheat lines, identifying 41 loci associated with plant height and lodging resistance, including 28 novel loci. Recently, the assembly of the reference genome of hexaploid oat provided a robust foundation for anchoring molecular markers to chromosomes, which enables the association of markers with gene functions [18]. A GWAS of lodging resistance in 126 European oat accessions has been conducted, and 13 candidate SNPs co-localized with plant height and lodging index have been identified [19]. However, additional phenotypic evaluations of lodging resistance-related traits in oats, coupled with sustained efforts to identify lodging-resistant loci, remain imperative, and lodging resistance-related genes have not yet been thoroughly characterized in oats.

In this study, 130 oat germplasm accessions were evaluated in two field trials. At the milk stage, GWAS was performed on seven lodging resistance-related traits: plant height (PH), fresh weight per stem (FWSS), length of the basal second internode (LBSI), diameter of the basal second internode (DBSI), wall thickness of the basal second internode (WTBSI), stem breaking strength (SBS), and stalk puncture strength (SPS). The objective was to identify genetic loci and candidate genes associated with lodging resistance, thereby providing theoretical insights into the molecular mechanisms of oat lodging resistance and supporting the breeding of new lodging-resistant cultivars.

## 2. Results

### 2.1. Phenotypic Data Analysis

**Figure 1:**
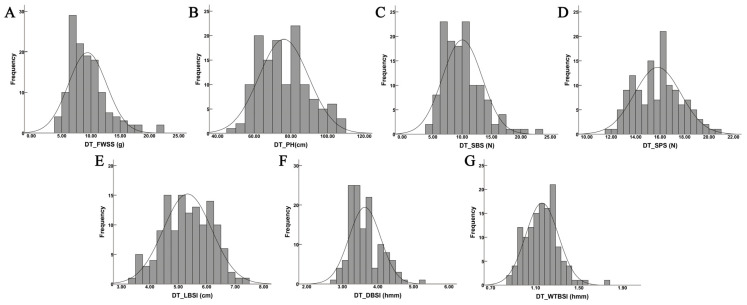
Frequency distribution for the seven lodging resistance-related traits in the DT site. Notes: (**A**) Fresh weight of single stem (FWSS), (**B**) plant height (PH), (**C**) stem breaking strength (SBS), (**D**) stalk puncture strength (SPS), (**E**) the length of basal second internode (LBSI), (**F**) the diameter of basal second internode (DBSI), (**G**) the wall thickness of basal second internode (WTBSI).

**Figure 2:**
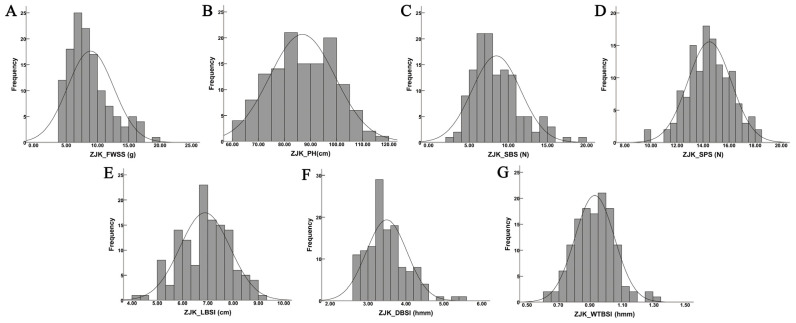
Frequency distribution for the seven lodging resistance-related traits in the ZJK site. Notes: (**A**) Fresh weight of single stem (FWSS), (**B**) plant height (PH), (**C**) stem breaking strength (SBS), (**D**) stalk puncture strength (SPS), (**E**) the length of basal second internode (LBSI), (**F**) the diameter of basal second internode (DBSI), (**G**) the wall thickness of basal second internode (WTBSI).

Seven lodging resistance-related traits, FWSS, PH, LBSI, DBSI, WTBSI, SBS, and SPS, were evaluated across 130 oat germplasm accessions in two environments. The results revealed considerable variation among the seven traits, with all coefficients of variation (CV) exceeding 10% (Table 1). Among them, SPS exhibited the lowest CV, whereas FWSS displayed the highest. SBS showed substantial variation at both experimental sites, with CV of 36.61% and 33.53%, respectively (Table 1). These results indicate that the lodging resistance-related traits possess abundant genetic diversity within this population. The heritabilities of lodging resistance-related traits were 0.43, 0.85, 0.39, 0.91, 0.34, 0.90, and 0.70, respectively (Table 1), which showed that the lodging resistance-related traits had high heritability. Moreover, the frequency distribution results showed that all seven traits exhibited continuous variation and approximately normal distributions, consistent with the inheritance pattern of quantitative traits (Figure 1 and Figure 2).

### 2.2. Correlation Analysis of Phenotypes

**Figure 3:**
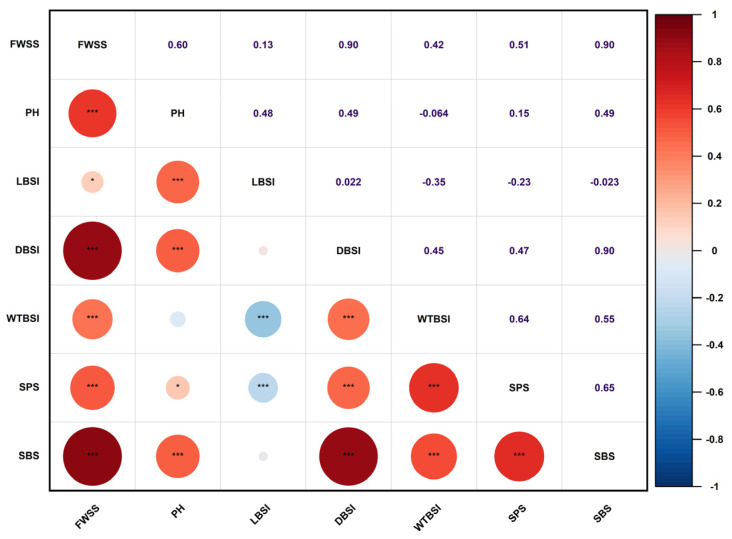
Correlation analysis among seven traits in 130 oat germplasm accessions. Notes: The numbers represent Pearson correlation coefficients (r) between pairs of traits. The circles visualize the correlations: red indicates positive correlations, while blue indicates negative correlations. The color intensity corresponds to the strength of the correlation, and circle size represents the r^2^ value, with larger circles indicating stronger correlations. * *p* ≤ 0.05, *** *p* ≤ 0.001.

The correlation analysis results between various traits showed that there was a close correlation between traits such as FWSS, PH, DBSI, SBS, and SPS in oat stalk lodging resistance (Figure 3). The correlation between PH, DBSI, WTBSI, SBS, and FWSS was 0.42–0.90, showing a significant positive correlation. Both DBSI and WTBSI demonstrated positive correlations with SBS and SPS; the correlation coefficient was 0.47–0.90. In contrast, LBSI showed significant negative correlations with WTBSI (r = −0.35) and SPS (r = −0.23), respectively.

### 2.3. SNP Distribution and Population Structure

**Figure 4:**
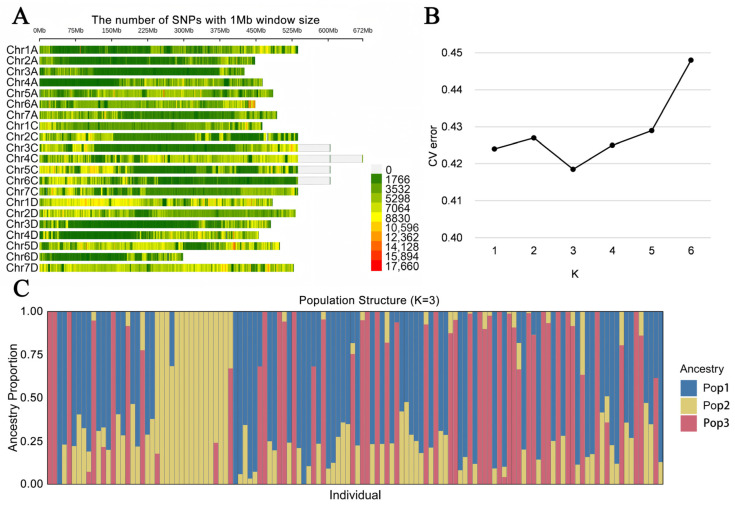
Analysis of population structure and SNP distribution in 130 oat samples. Notes: (**A**) SNP diversity analysis across the 21 chromosomes of oat, (**B**) plot of cross-validation error for K values ranging from 1 to 6, (**C**) grouping result based on K = 3.

To further elucidate the genetic basis of trait variation, SNP diversity was analyzed across the 130 oat germplasm accessions. A total of 36,928,068 SNPs were detected, spanning all 21 chromosomes of the oat genome. Among these, chromosome 4C contained the highest number of SNP markers, whereas chromosome 6D harbored the fewest (Figure 4A). Within the predefined model parameter range of K = 1 to 6, the cross-validation (CV) error initially decreased and then increased as K increased (Figure 4B). The minimum CV error (0.419) was observed at K = 3, indicating an optimal balance between model fit and complexity for the inferred population structure. Accordingly, the 130 oat accessions were clearly grouped into three genetically distinct subpopulations (Figure 4C). Thus, K = 3 was selected for the subsequent GWAS analyses.

### 2.4. Genome-Wide Association Analyses of Lodging Resistance Traits

**Figure 5:**
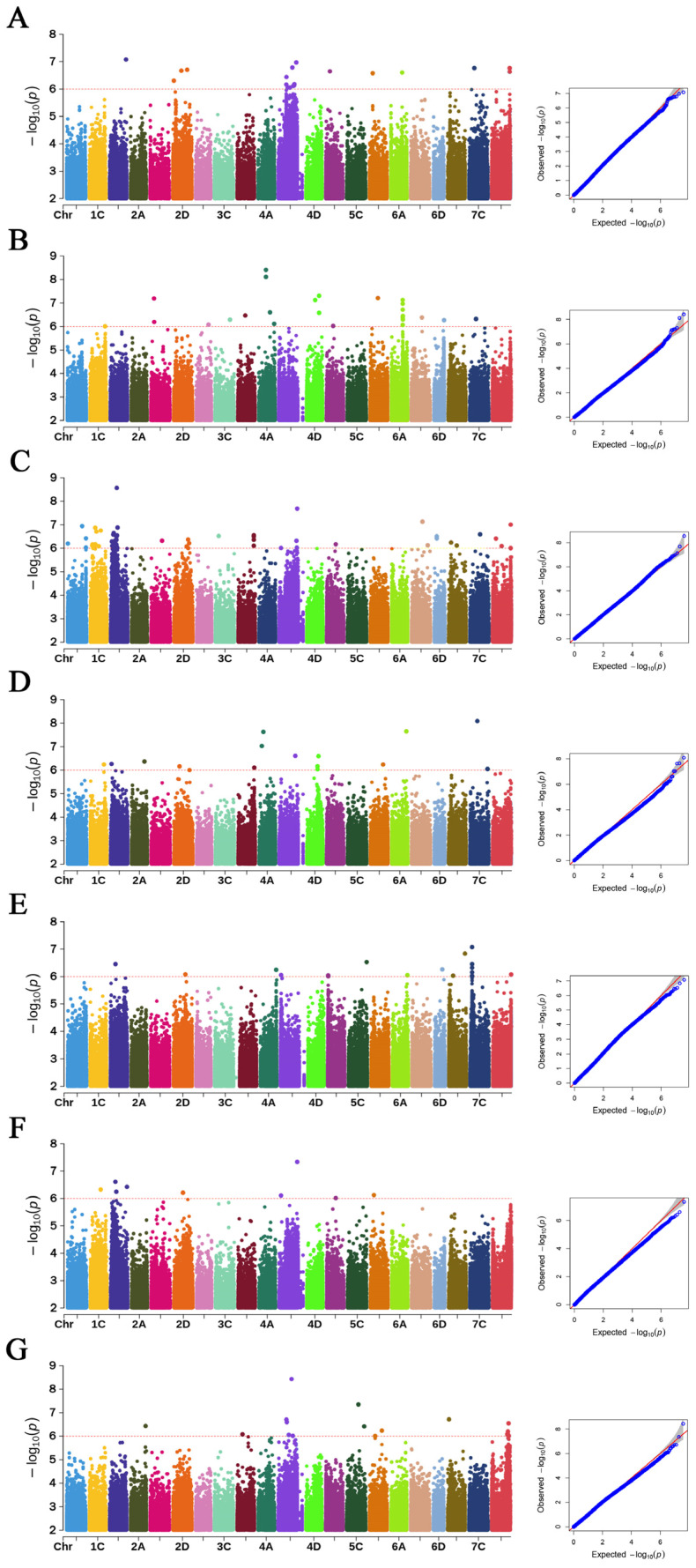
Manhattan plots and QQ plots for the seven lodging resistance-related phenotypic traits in the DT site. Notes: (**A**) Fresh weight of single stem (FWSS), (**B**) plant height (PH), (**C**) stem breaking strength (SBS), (**D**) stalk puncture strength (SPS), (**E**) the length of basal second internode (LBSI), (**F**) the diameter of basal second internode (DBSI), and (**G**) the wall thickness of basal second internode (WTBSI). Left panels show Manhattan plots for each trait, with the red dashed horizontal line marking the genome-wide significance threshold (−log_10_(P) = 6). Right panels show Q–Q plots comparing observed and expected −log_10_P values. The inflation patterns indicate effective control of population structure, with significant deviations at the upper tail reflecting true association signals.

**Figure 6:**
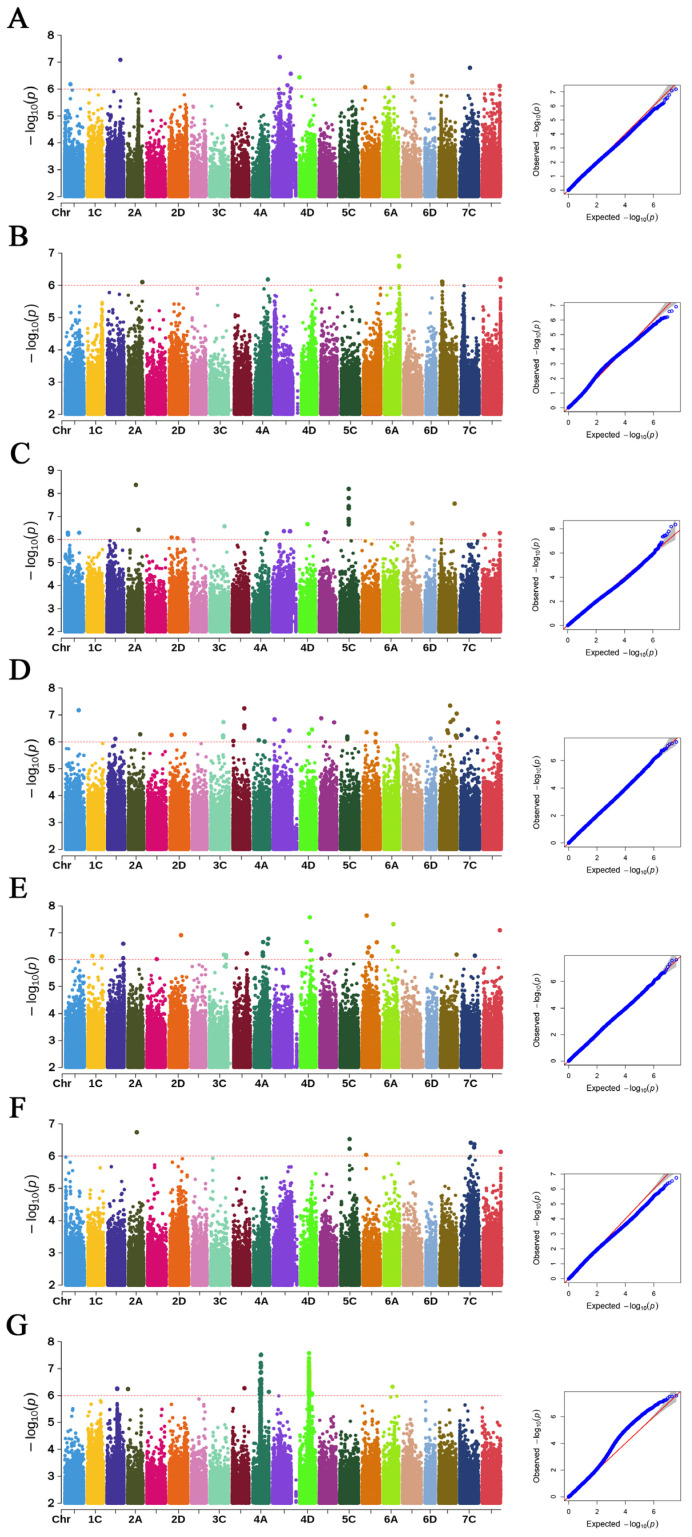
Manhattan plots and QQ plots for the seven lodging resistance-related phenotypic traits in the ZJK site. Notes: (**A**) Fresh weight of single stem (FWSS), (**B**) plant height (PH), (**C**) stem breaking strength (SBS), (**D**) stalk puncture strength (SPS), (**E**) the length of basal second internode (LBSI), (**F**) the diameter of basal second internode (DBSI), and (**G**) the wall thickness of basal second internode (WTBSI). Left panels show Manhattan plots for each trait, with the red dashed horizontal line marking the genome-wide significance threshold (−log_10_P = 6). Right panels show Q–Q plots comparing observed and expected −log_10_P values. The inflation patterns indicate effective control of population structure, with significant deviations at the upper tail reflecting true association signals.

A total of 379 significant QTNs were identified by GWAS across both environments, with the highest number (94) significantly associated with WTBSI and the fewest (18) significantly associated with DBSI (Supplementary Table S2). For PH, 38 significant QTNs were distributed across 16 chromosomes, with chromosome 6A harboring the most. Thirty-four QTNs correlated with FWSS were located on 12 chromosomes, mainly on chromosome 4C. Seventy-eight QTNs were associated with SBS across 19 chromosomes, while sixty-six QTNs were linked to SPS across 18 chromosomes, with the highest frequencies on chromosomes 7A (8) and chromosome 7C (13). Fifty-five LBSI-associated QTNs were identified across 18 chromosomes, predominantly on chromosome 4A (9), while eighteen DBSI-related QTNs were distributed over 12 chromosomes. The majority of WTBSI-associated QTNs were located on chromosomes 4A (16) and 4D (55) (Supplementary Table S2, Figure 5 and Figure 6).

Across the DT site, 160 significant QTNs associated with these seven traits were identified (Figure 5), distributed across all 21 chromosomes. The highest number of QTNs occurred on chromosome 4C (22), while chromosome 3A contained only one. Specifically, 26 QTNs were significantly associated with PH, 20 with FWSS, 48 with SBS, 20 with SPS, 18 with LBSI, 10 with DBSI, and 18 with WTBSI. At the ZJK site, GWAS identified 223 significant QTNs associated with these traits, with the greatest number detected on chromosome 4D (62). Of these, 12 QTNs were associated with PH, 16 with FWSS, 30 with SBS, 46 with SPS, 37 with LBSI, 8 with DBSI, and 76 with WTBSI (Figure 6).

Further comparative analysis of the two environments revealed four QTNs that were consistently detected in both environments (Table 2). Three of these common loci were associated with FWSS and located on chromosome 5D (chr5D_90334559) and chromosomes 7D (chr7D_504853535, chr7D_504853578). One shared locus associated with SBS was identified on chromosome 4C (chr4C_507724758). These overlapping loci may represent key genetic regions that play stable and critical roles in regulating lodging resistance-related traits across different environments.

### 2.5. Screening and Identification of Candidate Gene Analysis

For trait-associated loci exhibiting sharp peaks in the Manhattan plots, genes within a 20-kb interval upstream and downstream of the significant QTNs were annotated, resulting in the identification of 54 distinct candidate genes (Table 3). Five candidate genes were associated with PH, including members of the peptidase family, cytoplasmic aminopeptidase family, plastid acetyl-CoA carboxylase (ACC2), UDP-glycosyltransferase family, and serine/threonine protein phosphatase. For FWSS, five candidate genes were annotated across both environments, including those encoding MYM-type protein 1-like and endonuclease, although one annotated gene at the ZJK site lacked a clearly defined function.

Seven candidate genes were identified for SBS, associated with functions such as phosphoribosyl transferase, hydrolase, terpenoid synthase, ribonuclease H, and ascorbate iron-dependent oxidoreductase activity. Eleven candidate genes were annotated for SPS, linked to the WRKY30 transcription factor, E3 ubiquitin ligase, ascorbate iron-dependent oxidoreductase, and protein kinase activity. Fourteen candidate genes were implicated in LBSI, including those encoding ascorbate iron-dependent oxidoreductase, pentapeptide repeat domains, transporters, superoxide dismutase, YABB proteins, and glutathione S-transferase. No annotated genes were identified for the DBSI in either environment.

Twelve candidate genes were associated with WTBSI across both sites. Functional annotation revealed that these genes primarily encode peptidases, hydrolases, proteins with ribonuclease III activity, and 3’-exonuclease enzymes. Collectively, these co-annotated genes may play crucial regulatory roles in determining WTBSI by modulating pathways involved in cell wall metabolism.

## 3. Discussion

### 3.1. Phenotypic Analysis of Lodging Resistance Traits

In this study, statistical analysis of lodging resistance-related traits that exhibited a normal distribution across two environments revealed phenotypic variation rates exceeding 10% (Table 1) and indicated considerable diversity in these traits within the associated populations. Correlation analysis further uncovered close positive correlations between PH, DBSI, WTBSI, SBS, and FWSS (r = 0.42–0.90), underscoring the interconnectedness of morphological and mechanical traits in determining lodging risk. For instance, the significant positive associations of DBSI and WTBSI with SBS and SPS support the findings of Wang et al. [20], who reported that increasing stem diameter and wall thickness in the second internode is crucial for improving stem mechanical strength. Notably, while PH is widely recognized as a key contributor to lodging susceptibility [21], its strong positive correlation with SBS and diameter-related traits (DBSI, WTBSI) in the present oat panel suggests that taller lines may compensate for potential lodging risk through enhanced stem robustness. This stands in contrast to studies on wheat, where height and lodging resistance are often negatively correlated [22], highlighting species-specific differences in the relationships between these traits. Collectively, these results indicate that traits associated with stem thickness and mechanical strength are fundamental to reducing lodging susceptibility in oats.

### 3.2. Genome-Wide Association Analysis

Population structure is a critical factor affecting the reliability of GWAS results for complex traits. Accurate assessment of population structure can effectively correct false positive associations caused by genetic background heterogeneity [23]. In this study, the optimal model with K = 3, determined through 10-fold cross-validation, effectively minimized such interference, thereby ensuring the reliability of 379 significantly associated QTNs identified. This analysis also provided an essential foundation for accurately identifying stable loci associated with lodging resistance.

For GWAS of the seven lodging resistance-related traits, we employed the BLINK model, an enhanced version of Farm CPU, due to its superior statistical power in detecting minor-effect loci and improved computational efficiency compared to conventional GWAS models [24]. This analysis identified 379 significant QTNs distributed across all 21 chromosomes, which were associated with the target lodging resistance traits. Notably, our results are consistent with those of Peng et al. [25], who conducted Farm CPU-based GWAS on seven agronomic traits of forage oat and identified 94 significant loci and 155 candidate genes. Their study further revealed the dominant role of the D subgenome in key growth traits, the notable contribution of the C subgenome to leaf and spike traits, and the limited involvement of the A subgenome in only a few traits. Similarly, our study found that the number of lodging resistance-associated SNPs on the D subgenome 173 was significantly higher than that on the A 106 and C 104 subgenomes. From an evolutionary perspective, the D subgenome, which was incorporated later during oat polyploidization, may have retained more genes related to environmental adaptability. This finding enhances our understanding of the chromosomal distribution of lodging resistance loci in oats and provides a valuable direction for the targeted identification of resistance genes, particularly on the D and C subgenomes.

In addition, GWAS focused on lodging resistance traits in oats remain relatively scarce. Previous studies have mainly focused on agronomic traits such as plant height, panicle length, and stem length [26], whereas GWAS of lodging resistance-related traits including stem diameter, stem wall thickness, and stem bending resistance have been predominantly conducted in maize and rice. For instance, in rice, Meng et al. [27] identified 16 quantitative trait loci (QTL) associated with stem wall thickness. Our detection of 94 quantitative trait nucleotides (QTNs) for WTBSI suggests that stem wall thickness in oats is governed by a greater number of small-effect loci, consistent with the quantitative nature of this trait. In maize, Zhang et al. [28] identified 423 QTNs associated with stem diameter and bending strength using multi-locus GWAS, while our study detected 18 and 78 QTNs for DBSI and SBS, respectively. The lower number of QTNs identified in oats likely reflects the more complex genetic regulation of lodging resistance in this hexaploid species.

QTNs consistently detected across multiple environments are generally considered stable loci [29]. In this study, four co-localized QTNs (chr5D_90334559, chr7D_504853535, chr7D_504853578, and chr4C_507724758) were repeatedly identified across both environments. These environmentally stable QTNs likely represent key loci involved in stem lodging resistance and thus serve as promising candidates for further gene cloning and functional validation.

### 3.3. Functional Analysis of Candidate Genes

In this study, we screened candidate genes in the 20 kb flanking regions of 379 QTNs associated with oat lodging resistance traits. Combined with gene functional annotation, a total of 54 candidate genes with potential roles in regulating oat lodging resistance were ultimately identified; some of them were previously reported to be involved in key biological processes such as cell wall biosynthesis, lignin deposition, and hormone signaling, making them the top priority candidate genes. PH is associated with the UDP glycosyltransferase family. The family genes were shown to regulate lignin deposition in maize by mediating phenylpropanoid biosynthesis pathways, and its expression levels are significantly correlated with vascular bundle area and epidermal cell thickness in the stem [30]. In addition, FWSS is associated with a MYM-type zinc finger protein-related gene. Previous studies have confirmed that zinc finger MYM-type protein 1-like isoform X1 regulated node number and panicle weight, thereby indirectly improving lodging resistance [31,32]. Meanwhile, SPS was associated with both ubiquitin-related mechanisms and the WRKY30 transcription factor. E3 ubiquitin ligase GmILPA1 affects stem development and strength by regulating the gibberellin metabolic pathway [33]. WRKY30 directly regulates key monolignol biosynthesis genes and is involved in the genetic regulation of lignin biosynthesis [34]. LBSI is associated with one gene related to the YABB protein family. In rice, the homologous gene *OsYABBY4* negatively regulates the gibberellin signaling pathway, thereby inhibiting internode elongation and optimizing stem strength [35]. WTBSI is associated with a gene related to the GT31 family protein, which has been shown to localize to the Golgi apparatus and mediate primary and secondary cell wall cellulose biosynthesis in Arabidopsis (CAGE1 and CAGE2), with mutations in these genes leading to defective cell expansion and stunted growth [36]. These genes are considered to be reliable candidate genes for lodging resistance-related traits in oat, and further verification of their function will be helpful for further elucidating the underlying genetic and molecular mechanisms of yield-related traits. Meanwhile, we noted the novel observation of no annotated candidate genes for basal second internode diameter (DBSI), suggesting this trait may be regulated by non-coding genomic regions.

Although these findings enhance our understanding of the genetic architecture of lodging resistance, the functional annotation of candidate genes in oat remains insufficient compared with other crops. In addition, candidate gene screening in this study relied on reference genome annotation, and the completeness of the oat genome assembly may limit the accuracy of functional prediction. The 20 kb window size represents an empirical approximation; further refinement based on population-specific LD decay estimates and experimental validation, such as expression analysis or functional assays, will be critical to validate the roles of these candidate genes. Therefore, further comprehensive studies, including functional validation and co-expression network analysis, are needed to elucidate the molecular basis of stem lodging regulation and to provide a theoretical foundation for the genetic improvement of lodging resistance in oats.

## 4. Materials and Methods

### 4.1. Plant Materials and Growth Conditions

130 oat germplasm accessions were selected as materials from the National Crop Germplasm Resource Bank, the Clover Research Institute, Inner Mongolia Agricultural University, and the Institute of Animal Science, Chinese Academy of Agricultural Sciences. This group includes 115 germplasm materials and 15 improved cultivars. The germplasm information is described in Supplementary Table S1. Two experimental sites were established in Datong City (DT), Shanxi Province (112°34′~114°34′ E, 39°03′~40°44′ N), and Zhangjiakou City (ZJK), Hebei Province (41°28′24″ N, 115°1′3″ E). At the DT site, the oat germplasms were sown on 24 April 2023. At the ZJK site, they were sown on 16 May 2023. A randomized block design with three replications was employed at both experimental sites. Each accession was planted in a single 1.5 m row with a row spacing of 60 cm and an intra-row spacing of 5 cm, resulting in 30 plants per accession. Sowing was performed manually at a depth of 3–5 cm. Pests, diseases, and weeds were managed with appropriate pesticides as needed throughout the growing seasons.

### 4.2. Phenotypic Data Collection and Analysis

The milk stage refers to the period after oats flower and before maturity. During this stage, the plant has its highest center of gravity, and the panicle is at its maximum weight, making it the critical period for lodging resistance assessment. Therefore, lodging-related phenotypic traits, including PH, FWSS, LBSI, DBSI, WTBSI, SBS, and SPS, were measured at the milk stage [37]. Five representative plants of each line from each replicate were randomly selected from each accession to measure PH, FWSS, LBSI, DBSI, WTBSI, SBS, and SPS. The mean values for each line were computed for each trait. Plant height (PH) was measured using a ruler from plant base to the tip of spikes under natural state. The basal second internode (LBSI) was the second internode upward from the ground, and the lengths were measured using a ruler. The diameter of the basal second internode (DBSI) was measured at the midpoint using a digital caliper. Subsequently, the internode was carefully split longitudinally to measure the wall thickness of the basal second internode (WTBSI) directly at the same position. The main stems of plants were randomly sampled by cutting at ground level, with specimens containing stems, leaves, sheaths, and spikes. The fresh weight of a single stem (FWSS) was immediately determined using an electronic balance [38]. Both stalk puncture strength (SPS) and stem breaking strength (SBS) were measured with a Stem Strength Tester (YYD-1; The Zhejiang Top Instrument Co., Ltd., Hangzhou, China) using internode segments of uniform length [39]. At the base of the stem, the middle part of the second and third internodes was inserted at a constant speed and perpendicular to the direction of the stem, and the maximum penetration of the stem epidermis was read. Similarly, the stem breaking strength was also pressed at the center of the stalk at a uniform speed, and the value was recorded [29].

Microsoft Excel 2019 was used to organize and preprocess the phenotypic data. IBM SPSS Statistics 26.0 software (SPSS, Inc., Chicago, IL, USA) was employed to calculate the mean, kurtosis, skewness, standard deviation (SD), and coefficient of variation (CV = SD/mean) [40] for each phenotypic indicator, as well as to generate frequency distribution plots. Correlation analyses were performed using Origin 2024 software. The Broad-sense heritability (H^2^) was calculated for kernel color traits according Nyquist as H^2^  =  δ^2^_G_/(δ^2^_G_  +  δ^2^_E_/n) where δ^2^_G_ and δ^2^_E_ are genetic variance and residual variance, respectively [41].

### 4.3. SNP Calling and Population Structure

Fresh leaf tissues were collected from the field in equal quantities. Samples were immediately placed into pre-chilled centrifuge tubes, sealed, and rapidly frozen in liquid nitrogen. Genomic DNA was extracted using the Plant Genomic DNA Extraction Kit (DP305; Beijing Tian gen Biochemical Co., Ltd., Beijing, China).

High-quality DNA samples were then used for genomic library construction and high-throughput sequencing on the Illumina NovaSeq 6000 platform, yielding approximately 59.4 GB of raw sequencing data per genotype.

During raw data preprocessing, Trimmomatic v0.39 was first employed to perform stringent quality control, removing low-quality reads, sequencing adapters, and primer sequences to obtain high-quality clean data. The filtered reads were subsequently aligned to the oat reference genome (https://wheat.pw.usda.gov/GG3/graingenes_downloads/oat-ot3098-pepsico, accessed on 12 November 2024) using BWA-MEM v0.7.17 software [42], and SNP calling was conducted with GATK Haplotype Caller (v4.2.3.062) [43]. In parallel, an additional SNP-calling pipeline was performed using SAMtools (v1.13) mpileup combined with Var Scan (v2.4.6) to improve variant detection accuracy. Finally, VCF tools (v0.1.16) was used to filter SNPs with a missing rate > 10%, minor allele frequency (MAF) < 0.05, or minimum sequencing depth < 5, resulting in 36,928,068 high-quality SNPs distributed across 21 oat chromosomes for subsequent GWAS [44].

The “CM plot” R (v4.1.3) package was used to generate genome-wide SNP density plots, to visualize SNP distribution across chromosomes. Population structure analysis was performed using Admixture software (v1.3.0) with K values ranging from 1 to 6. The optimal K value was based on the minimum cross-validation (CV) error.

### 4.4. Genome-Wide Association Study

Genome-wide association analyses were conducted using the Bayesian-information and Linkage-disequilibrium Iteratively Nested Keyway (BLINK) model in the GAPIT3 (v3.1.0) package. This approach enhances statistical power and computational speed [24]. A significance threshold of −log_10_(P) ≥ 6 (LOD ≥ 6) was applied to identify significant association signals. GWAS results were visualized using the R package “CM plot” [45], which allows for intuitive illustration of the genomic distribution and statistical significance of associated loci. Manhattan plots and quantile-quantile (Q-Q) plots were generated to depict these GWAS results.

### 4.5. Candidate Gene Identification

Based on the above GWAS results, markers exceeding the significance threshold in both environments were selected. Statistical analyses were then conducted on these markers to extract information on significant QTNs associated with target traits. Candidate genes were identified within a 20 kb range upstream and downstream of each significant QTN (total of 40 kb) based on the oat reference genome (OT3098) annotation file. This screening interval was established by referring to the widely adopted strategy in genome-wide association studies of forage crops and fully considering the linkage disequilibrium (LD) decay characteristics of oat genomes [46], which ensures effective coverage of functional genes in LD with significant SNPs while avoiding the inclusion of excessive irrelevant genes that would increase the difficulty of subsequent functional validation. Functional annotation of these genes was subsequently performed using the Egg NOG-mapper online tool (http://eggNOG-mapper.embl.de, accessed on 15 January 2025), enabling the identification of candidate genes potentially involved in lodging resistance in oat.

## 5. Conclusions

In this study, 36,928,068 high-quality SNPs were used to conduct genome-wide association analysis on 130 forage oat germplasm accessions using the BLINK model, and a total of 379 significantly associated QTNs were screened under two environments. Subsequent candidate gene screening and functional annotation yielded 54 genes linked to lodging resistance in oats. Functional annotation suggested that these genes may collectively contribute to lodging resistance by regulating cell wall metabolism, stem development, mechanical strength, and biomass allocation pathways. These findings provide valuable genetic resources and efficient molecular markers for the genetic improvement of lodging resistance in oat, thereby facilitating the breeding of new lodging-resistant varieties.

## Figures and Tables

**Table 1:** Descriptive statistics for oat lodging resistance traits in two environments.

Traits	En	Range	Mean ± SD	Skewness	Kurtosis	CV%	H^2^
FWSS (g)	DT	3.76–21.80	9.41 ± 3.27	1.13	2.33	34.78%	0.42
	ZJK	4.07–26.48	8.90 ± 3.67	1.52	3.54	41.27%	
PH (cm)	DT	49.28–108.38	76.14 ± 13.47	0.40	−0.54	17.69%	0.85
	ZJK	62.00–117.44	86.87 ± 12.56	0.03	−0.77	14.46%	
LBSI (cm)	DT	3.31–7.33	5.33 ± 0.85	−0.10	−0.66	16.01%	0.39
	ZJK	4.14–9.17	6.89 ± 0.99	−0.18	−0.22	14.37%	
DBSI (mm)	DT	2.71–5.26	3.63 ± 0.45	0.81	0.57	12.25%	0.91
	ZJK	2.62–5.55	3.49 ± 0.54	0.91	1.36	15.36%	
WTBSI (mm)	DT	0.86–1.75	1.16 ± 0.15	0.51	0.97	13.04%	0.34
	ZJK	0.62–1.30	0.92 ± 0.13	0.20	0.23	13.76%	
SBS (N)	DT	4.71–23.42	10.04 ± 3.36	1.10	1.73	33.53%	0.90
	ZJK	2.78–19.09	8.48 ± 3.10	0.89	0.69	36.61%	
SPS (N)	DT	11.78–20.74	15.81 ± 1.90	0.19	−0.56	12.02%	0.70
	ZJK	9.88–18.24	14.50 ± 1.67	−0.08	−0.05	11.50%	

En stands for environment. SD stands for standard deviation. CV stands for coefficient of variation. H^2^ stands for broad sense heritability. PH, FWSS, LBSI, DBSI, WTBSI, SBS, and SPS stand for plant height, the fresh weight of single stem, the length of basal second internode, the diameter of basal second internode, the wall thickness of basal second internode, the stem breaking strength, and stalk puncture strength, respectively. DT stands for Da Tong City. ZJK stands for Zhangjiakou City.

**Table 2:** Co-located QTN locus information.

Traits	Marker	Chromosome	Position	*p*-Value_DT	*p*-Value_ZJK
FWSS	chr5D_90334559	5D	90,334,559	2.65 × 10^−7^	8.57 × 10^−7^
	chr7D_504853535	7D	504,853,535	2.36 × 10^−7^	7.59 × 10^−7^
	chr7D_504853578	7D	504,853,578	1.73 × 10^−7^	7.84 × 10^−7^
SBS	chr4C_507724758	4C	507,724,758	2.06 × 10^−8^	4.36 × 10^−7^

**Table 3:** Annotation of candidate genes for SNP loci associated with lodging resistance in oat.

Trait	En	Chr.	Start	End	Gene ID	Gene Function
PH	DT	4A	319,007,360	319,011,287	AVESA.00001b.r3.4Ag0002063.1	Peptidase family
		6A	314,051,169	314,054,439	AVESA.00001b.r3.6Ag0001844.1	Cytosol aminopeptidase family
	ZJK	6A	420,589,269	420,603,312	AVESA.00001b.r3.6Ag0003033.1	Plastid acetyl-CoA carboxylase (ACC2)
		7D	505,012,188	505,014,273	AVESA.00001b.r3.7Dg0003250.1	UDP-glycosyltransferase family protein
		7D	506,576,675	506,580,544	AVESA.00001b.r3.7Dg0003286.1	Serine/threonine-protein phosphatase
FWSS	DT	2D	25,371,852	25,375,270	AVESA.00001b.r3.2Dg0000217.1	Zinc finger MYM-type protein 1-like
		4C	227,477,938	227,481,812	AVESA.00001b.r3.4Cg0002039.1	Core-2/I-Branching enzyme
		4C	231,853,602	231,858,240	AVESA.00001b.r3.4Cg0002062.1	Plastid RNA processing protein involved in chloroplast development and biogenesis
		6A	314,373,548	314,374,445	AVESA.00001b.r3.6Ag0001847.1	Endo chitinase
	ZJK	1D	389,035,273	389,039,059	AVESA.00001b.r3.1Dg0002486.1	-
SBS	DT	1A	404,722,032	404,726,986	AVESA.00001b.r3.1Ag0002435.1	Anthranilate phosphoribosyl transferase
		3C	93,761,443	93,764,150	AVESA.00001b.r3.3Cg0000843.1	Alpha/beta hydrolase family
		7A	68,995,610	69,004,371	AVESA.00001b.r3.7Ag0000828.1	Ribonuclease H protein
		7D	507,124,494	507,127,340	AVESA.00001b.r3.7Dg0003299.1	Threonine-dependent oxidoreductase family protein
	ZJK	1A	409,699,603	409,707,403	AVESA.00001b.r3.1Ag0002520.1	Terpene synthase family protein (NES)
		2D	242,124,014	242,136,421	AVESA.00001b.r3.2Ag0000690.1	Protein desumoylation
		2D	238,006,510	238,014,542	AVESA.00001b.r3.2Dg0002662.1	Calponin homology domain
SPS	DT	3D	451,748,925	451,751,087	AVESA.00001b.r3.3Dg0002508.1	Basic region leucine zipper
		5D	355,657,434	355,658,796	AVESA.00001b.r3.5Dg0001797.1	WRKY transcription factor (WRKY30)
	ZJK	3D	9,300,350	9,302,877	AVESA.00001b.r3.3Dg0000102.1	Protein of unknown function
		4C	38,215,958	38,221,817	AVESA.00001b.r3.4Cg0000490.1	Cob N/Magnesium Chelatase (CHLH)
		4C	38,221,968	38,224,853	AVESA.00001b.r3.4Cg0000491.1	STELLO glycosyl transferases
		5A	37,913,523	37,917,195	AVESA.00001b.r3.5Ag0000489.1	Phosphoinositide phospholipase C
		5A	37,945,313	37,948,801	AVESA.00001b.r3.5Ag0000490.1	Iron ascorbate-dependent oxidoreductase family protein
		5D	365,643,406	365,645,826	AVESA.00001b.r3.5Dg0001898.1	RING-type E3 ubiquitin transferase
		7A	486,025,311	486,026,446	AVESA.00001b.r3.7Ag0002909.1	Protein kinase activity
		7A	488,980,641	488,990,316	AVESA.00001b.r3.7Ag0002943.1	TRAFAC class myosin-kinesin ATPase superfamily protein.
		7A	488,990,567	489,000,788	AVESA.00001b.r3.7Ag0002944.1	ABC transporter superfamily protein.
LBSI	DT	4A	447,360,143	447,361,610	AVESA.00001b.r3.4Ag0003694.1	F-box associated domain
		5A	16,543,733	16,548,785	AVESA.00001b.r3.5Ag0000226.1	Nonaspanins (TM9SF) (TC9.A.2) family protein
		5C	514,065,428	514,066,914	AVESA.00001b.r3.5Cg0002225.1	Iron ascorbate-dependent oxidoreductase family (ACO1) protein
		5C	514,083,463	514,088,461	AVESA.00001b.r3.5Cg0002226.1	Protein disulfide isomerase family (PDIL2-3)
		7A	117,149,119	117,161,924	AVESA.00001b.r3.7Ag0001416.1	Pentatricopeptide repeat domain
		7A	117,152,033	117,156,539	AVESA.00001b.r3.7Ag0001417.1	EXS family
		7C	55,775,943	55,781,673	AVESA.00001b.r3.7Cg0000821.1	CorA-likeMg^2+^ transporter protein
		7D	520,027,020	520,030,018	AVESA.00001b.r3.7Dg0003526.1	Superoxide dismutase (FSD2)
	ZJK	1D	458,224,314	458,226,924	AVESA.00001b.r3.1Dg0003327.1	Lycopene cyclase protein
		4A	252,869,967	252,876,479	AVESA.00001b.r3.4Ag0001056.1	YABBY protein
		4A	418,580,808	418,591,505	AVESA.00001b.r3.4Ag0003345.1	Helicase family
		6A	298,157,684	298,160,246	AVESA.00001b.r3.6Ag0001631.1	Glutathione S-transferase
		6A	425,223,257	425,228,039	AVESA.00001b.r3.6Ag0003091.1	Chaperonin (HSP60) family protein
		7A	465,467,447	465,469,075	AVESA.00001b.r3.7Ag0002791.1	Protein kinase domain
WTBSI	DT	4C	375,950,135	375,960,349	AVESA.00001b.r3.4Cg0002505.1	Peptidase M24B family protein
		7D	455,942,111	455,954,377	AVESA.00001b.r3.7Dg0002586.1	Plant protein of unknown function
		7D	473,845,493	473,849,453	AVESA.00001b.r3.7Dg0002842.1	Glycosyl transferase 31 family protein
	ZJK	1D	291,079,730	291,083,458	AVESA.00001b.r3.1Dg0001192.1	Dienelactone hydrolase family
		3D	347,440,640	347,443,676	AVESA.00001b.r3.3Dg0001525.1	Ribonuclease III activity
		4D	262,753,531	262,760,073	AVESA.00001b.r3.4Dg0001217.1	Fts J-like methyl transferase
		4D	268,863,762	268,869,296	AVESA.00001b.r3.4Dg0001265.1	Cycloeucalenol cycloisomerase
		4D	268,893,661	268,895,312	AVESA.00001b.r3.4Dg0001266.1	Class I-like SAM-binding methyltransferase superfamily
		4D	269,750,629	269,752,463	AVESA.00001b.r3.4Dg0001274.1	Universal ribosomal protein family
		4D	269,835,442	269,840,109	AVESA.00001b.r3.4Dg0001277.1	3’exoribonuclease family
		4D	269,931,907	269,933,471	AVESA.00001b.r3.4Dg0001281.1	Peptidase C1family
		4D	272,120,828	272,126,959	AVESA.00001b.r3.4Dg0001303.1	NPH3 family protein

En stands for Environment. Chr stands for Chromosome.

## Data Availability

Upon reasonable request, the datasets used and/or analyzed in this study are available from the corresponding author.

## Supplementary Materials

The following supporting information can be downloaded at: https://www.mdpi.com/article/10.3390/plants15060861/s1, Table S1: Information on 130 oat germplasm resources; Table S2: Two environments, 130 oat germplasm resources, 7 lodging-related traits, 379 significant QTNs identified.

## Abbreviations

The following abbreviations are used in this manuscript:

GWAS	genome-wide association study
BLINK	Bayesian-information and Linkage-disequilibrium Iteratively Nested Keyway
SNP	single-nucleotide polymorphism
QTN	quantitative trait nucleotide
QTL	quantitative trait loci
PH	plant height
FWSS	the fresh weight of single stem
LBSI	the length of basal second internode
DBSI	diameter of basal second internode
WTBSI	wall thickness of basal second internode
SBS	stem breaking strength
SPS	stalk puncture strength
DT	Da Tong City
ZJK	Zhangjiakou City
